# A Case Report of a Patient With Renal Artery Thrombosis Despite Being on Treatment Dose of Direct Oral Anticoagulant

**DOI:** 10.7759/cureus.101885

**Published:** 2026-01-20

**Authors:** Mahmoud Hatem Mohamed Mohamed Elwasif, Mohamed Elshenawy, Ali Javeed, Ramadan Ahmed

**Affiliations:** 1 General Internal Medicine, Scarborough General Hospital, Scarborough, GBR; 2 Geriatrics, Scarborough General Hospital, Scarborough, GBR; 3 Internal Medicine, Scarborough General Hospital, Scarborough, GBR

**Keywords:** atrial fibrillation, direct oral anticoagulants (doac), infective endocarditis, renal artery thrombosis, splenic infarcts

## Abstract

Renal artery thrombosis is a rare but serious cause of acute abdominal pain, which can rapidly progress to renal infarction. It is difficult to diagnose as it is very rare and can present in a similar manner to other causes of abdominal pain. We present the case of a 76-year-old man who developed right renal artery thrombosis leading to a renal infarction in addition to a splenic infarct. This happened despite being on a treatment dose of a direct oral anticoagulant (DOAC) in the presence of *Staphylococcus haemolyticus* bacteremia. Despite medical management including therapeutic anticoagulation, intravenous (IV) antibiotics and IV fluids, his condition deteriorated, and he died due to *S. haemolyticus* sepsis. This case highlights the complex interaction between sepsis, atherosclerosis, thrombosis and the diagnostic challenges posed by such a rare condition.

## Introduction

Renal artery thrombosis is an extremely rare diagnosis with an estimated incidence of 14/1000 in autopsies and 0.02/1000 among emergency department presentations [1,2]. There are far more common causes of acute abdominal pain, including renal colic [3] and acute cholecystitis [4]. Additionally, the patient had a past history of chronic pancreatitis, which can present with abdominal pain. Given that the patient was receiving a treatment dose of direct oral anticoagulants (DOACs) for atrial fibrillation, the likelihood of thrombosis is further reduced, as DOACs are well established to lower the risk of thromboembolism [5]. Perhaps one explanation is that it happened due to a hypercoagulable state caused by infection, as sepsis is known to increase the risk of thrombosis [6]. Endocarditis due to *Staphylococcus haemolyticus* is rare [7] but can happen in patients with prosthetic valves [8].

This report describes a case of right renal artery thrombosis with splenic infarct in a patient with *S. haemolyticus* sepsis and a past history of transcatheter aortic valve implantation (TAVI).

## Case presentation

A 76-year-old man was admitted to Scarborough General Hospital with a general practitioner (GP) referral due to a two-day history of gradual-onset, intermittent epigastric and right upper quadrant (RUQ) abdominal pain radiating to the back. He had one episode of vomiting. Examination showed tenderness and guarding in the epigastrium and RUQ and right lower quadrant.

He had a past medical history of atrial fibrillation for which he was taking edoxaban 30 mg per day, transcatheter aortic valve implantation (TAVI), heart failure, chronic pancreatitis, type 2 diabetes mellitus and ischaemic heart disease (IHD), including myocardial infarction (MI) and cardiac bypass.

He had a CT of the abdomen-pelvis with contrast on the same day of his admission, which was reported as follows: there is no enhancement of the right kidney with sparing of the upper pole. There is a focal hyperdensity within the right distal main renal artery. There is no hydroureteronephrosis. The liver is heterogeneous with periportal oedema. There is no portal venous thrombosis. The spleen is heterogeneous with a focus of non-enhancement laterally, which could represent an infarct. There is significant aortoiliac atheromatous disease.

The reporting radiologist's impression was that it showed a right renal infarct with a possible thrombus within the right renal artery in addition to a likely focal splenic infarct. Additionally, it showed a background significant atheromatous disease.

**Figure 1:**
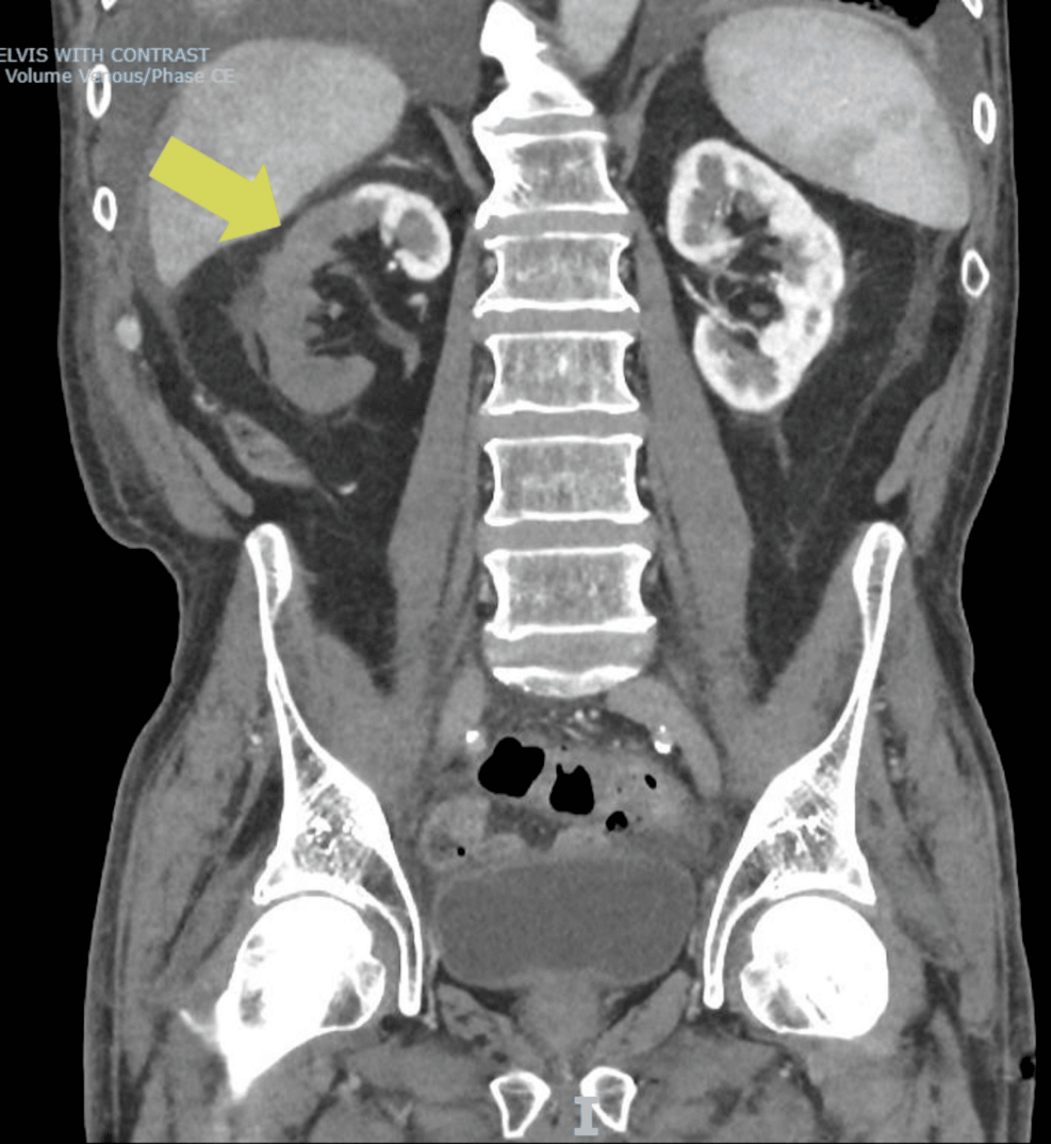
A lack of contrast uptake in the right kidney compared to the left kidney

**Figure 2:**
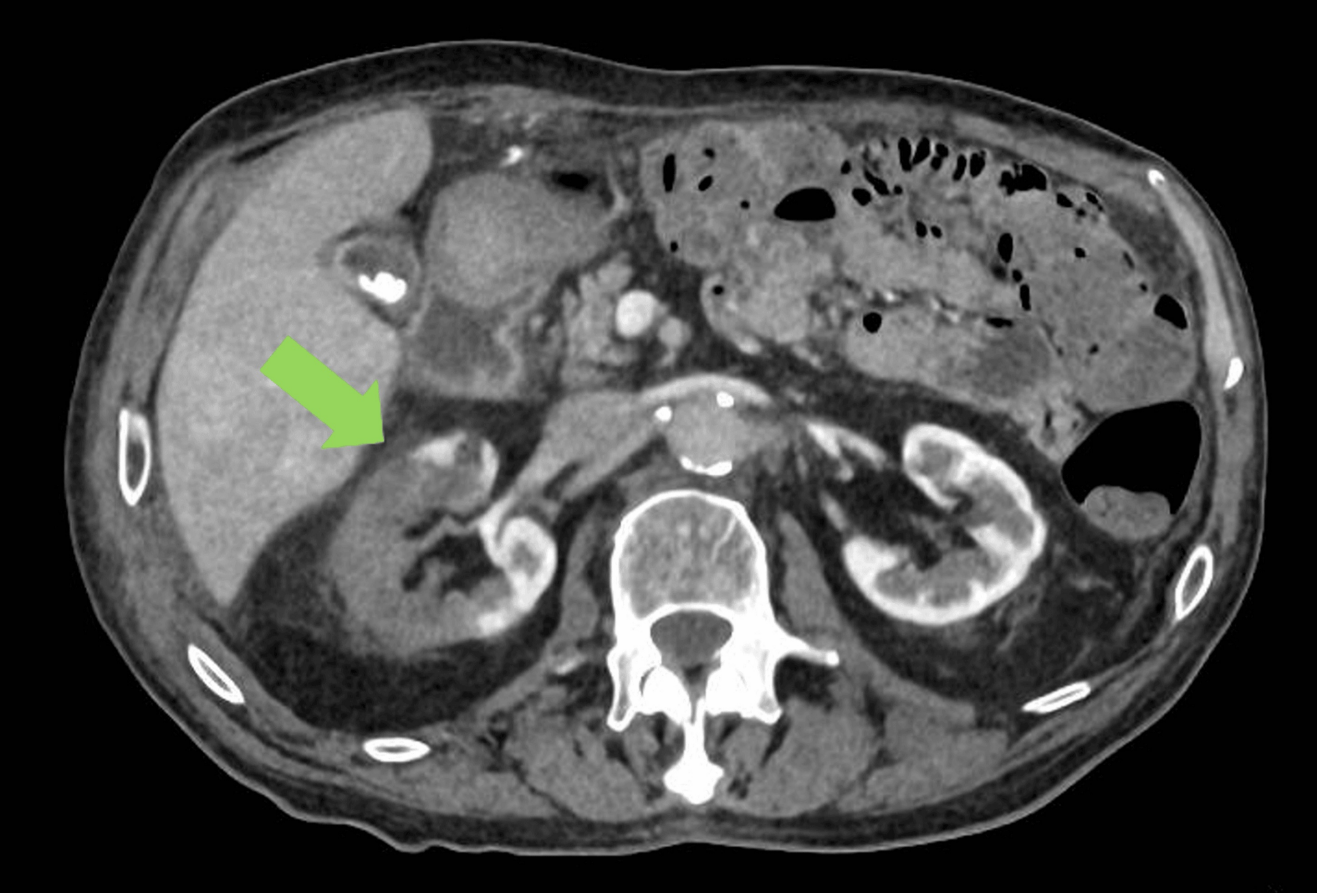
Contrast uptake in the area supplied by the right accessory renal artery rather than the right renal artery due to thrombosis

**Figure 3:**
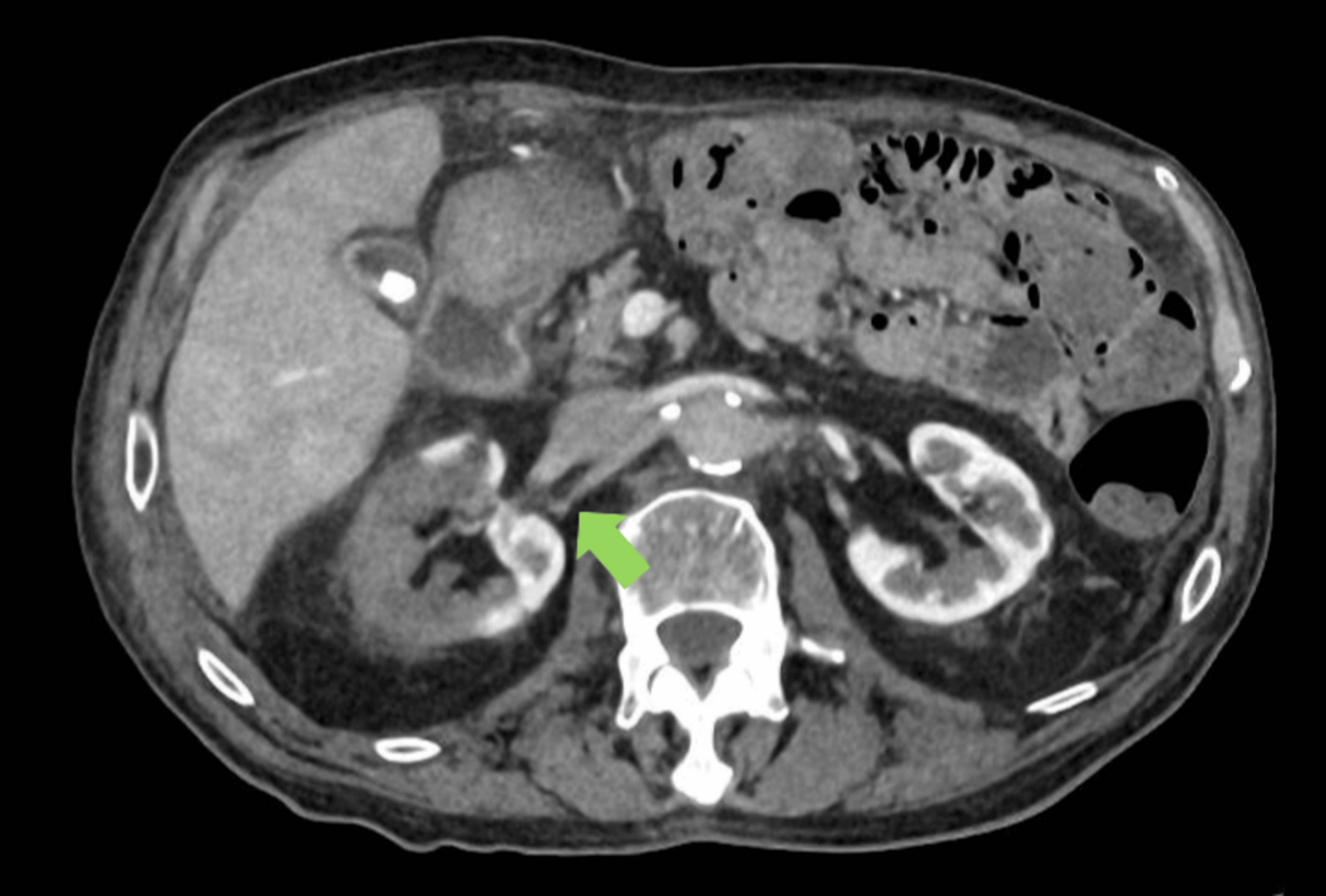
Further demonstration of the effect of right renal artery thrombosis

**Figure 4:**
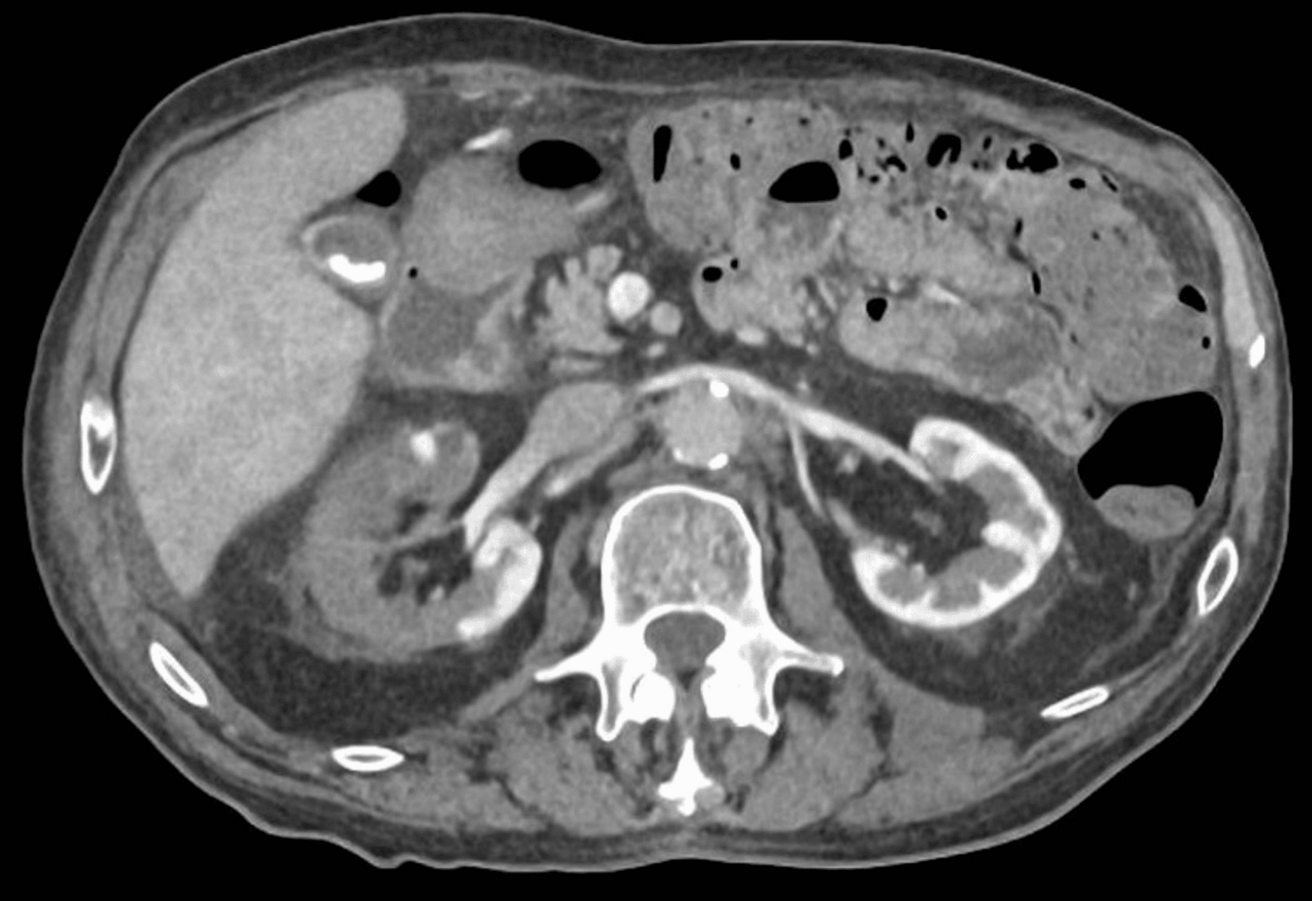
More imaging of the renal vessels, including the right renal vein, which did not show evidence of a thrombus

This scan was then further reviewed by a radiology consultant locally within the trust, who reported the following: as reported, there is an infarct of most of the right kidney due to the occlusion of the renal artery. An accessory right renal artery is patent, supplying the upper pole to some extent. The left kidney enhances well. The study does not have an arterial phase, and a subtle abnormality within the descending thoracic and abdominal aorta cannot be ruled out, but there is generalised atherosclerosis (Figures 1-4).

**Figure 5:**
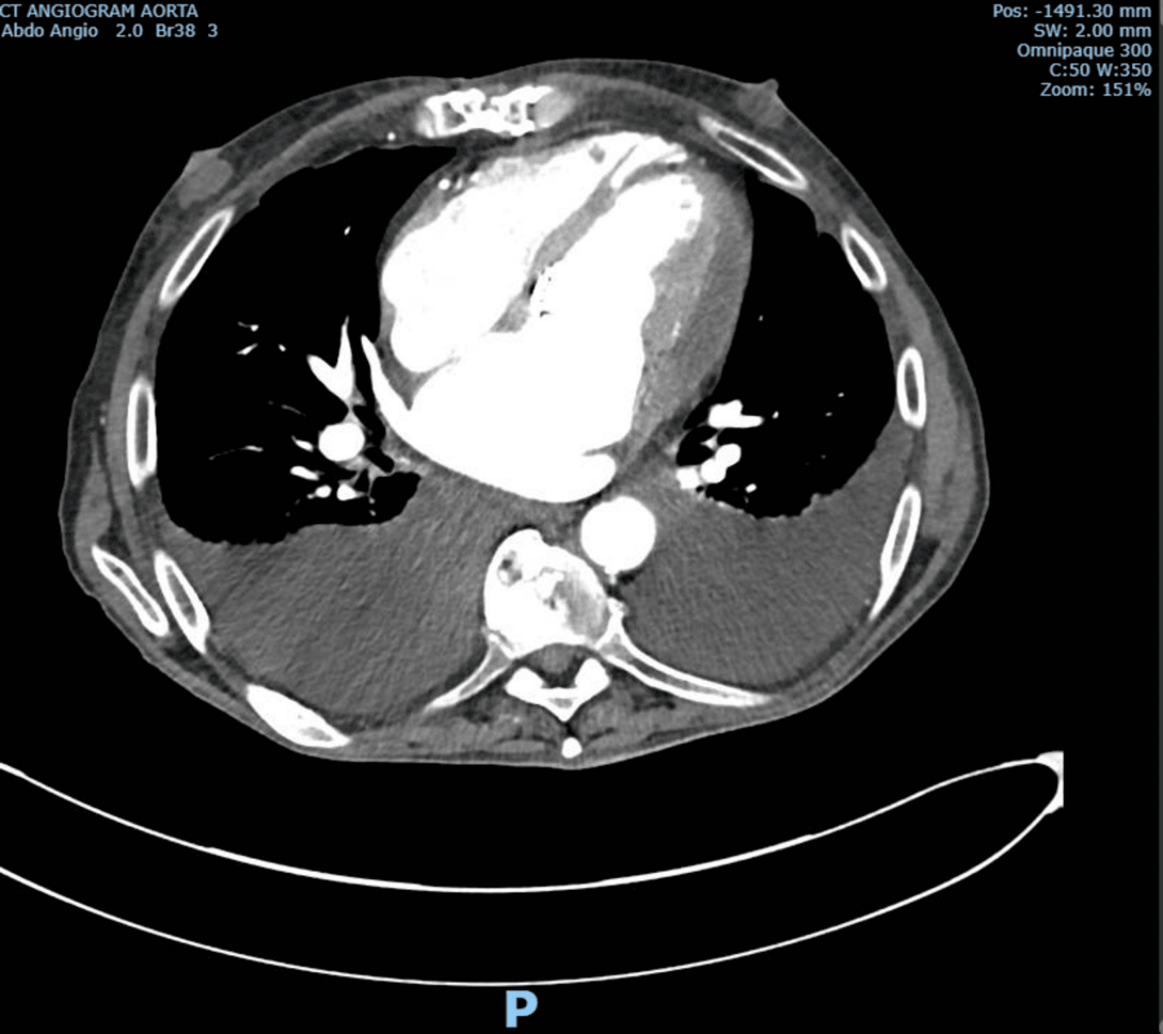
Substantial dense pericardial effusion (suspected hemopericardium)

A further CT-gated scan of the thoracic aorta was done the next day. It was reported as follows: no source of thrombus or embolus is demonstrated. The echo assessment of the TAVI should be considered if it is suspected as a source. There is a substantial, dense pericardial effusion, which may be hemopericardium. The apical myocardium is rather thin; has there been an MI previously that may have affected its integrity (Figure 5)?

The reporting radiology consultant discussed with a consultant vascular surgeon, and it was agreed that in view of the renal infarct, there is no benefit to attempting the recanalisation of the renal artery either endovascularly or surgically. It was also agreed that he is not fit for surgery. Therefore, he was treated with a therapeutic dose of enoxaparin after discussion with the haematology team.

He denied any issues with adherence to his edoxaban.

The blood tests showed evidence of infection (Table 1), which was initially treated with intravenous (IV) broad-spectrum antibiotics. However, on the fourth day of his admission, blood cultures showed *Staphylococcus haemolyticus* in all four bottles. Therefore, the antibiotics were changed after discussion with the on-call microbiology consultant. Additionally, the blood tests revealed evidence of acute kidney injury (AKI) on top of known chronic kidney disease (CKD) (AKI on CKD), which was initially treated with IV fluids, but as it continued worsening to AKI stage 3, he was transferred to York Hospital under renal team for further management and tighter optimisation of fluid status five days after his admission. Furthermore, the blood tests were negative for possible autoimmune causes of thrombosis, including antiphospholipid syndrome.

**Table 1:** Blood tests Ab, antibodies; ANA, antinuclear antibody; ACA, anticardiolipin antibody; B2, beta 2; APTT, activated partial thromboplastin time; CENTH, anticentromere antibodies; DNAH, anti-double-stranded DNA antibody; JO1H, anti-Jo-1 antibodies; RIBPH, anti-ribosomal P antibody; RNPH, antinuclear ribonucleoprotein (anti-nRNP) antibody; RO52H, anti-Ro52 antibody; SSAH, anti-Sjögren's-syndrome-related antigen A (SSA) (anti-Ro) autoantibodies; SMH, anti-Smith antibodies; S70AH, anti-Scl-70 (anti-topoisomerase I) antibody; SSBH: anti-La (anti-SSB) antibody

Investigation	Value	Reference Range
C4 Complement, Serum (G/L)	0.24	0.10-0.40
C3 Complement, Serum (G/L)	1.19	0.90-1.80
Immunoglobulin M, Serum (G/L)	1.27	0.50-2.00
Anti-proteinase 3 Ab (CU)	<2.3	0.0-19.9
ANA CENTH (U)	<0.5	0.0-14.9
ANA DNAH (IU/mL)	7.7	0.0-26.9
ANA JO1H (U)	<0.3	0.0-9.9
ANA RIBPH (U)	<0.3	0.0-9.9
ANA RNPH (U)	<0.5	0.0-29.9
ANA RO52H (U)	0.5	0.0-29.9
ANA SSAH (U)	2.5	0.0-29.9
ANA S70AH (U)	<0.5	0.0-29.9
ANA SMH (U)	<0.3	0.0-4.9
ANA SSBH (U)	0.5	0.0-29.9
B2-Glycoprotein (CU)	15.2	0.0-19.9
ACA Antiphospholipid Ab (CU)	11.8	0.0-19.9
Anti-myeloperoxidase Ab (CU)	<3.2	0.0-15.9
Haemoglobin (G/L)	101	130-180
White Cell Count (×10^9^/L)	16.28	4.00-11.00
Platelet Count (×10^9^/L)	137	150-450
Red Cell Count (×10^12^/L)	3.33	4.30-5.80
Mean Cell Volume (fL)	85	80-100
Mean Cell Haemoglobin (Pg)	30.3	27.0-32.0
Haematocrit (L/L)	0.284	0.390-0.500
Immature Granulocytes (×10^9^/L)	0.12	0.00-0.20
Neutrophils (×10^9^/L)	14.37	2.00-8.00
Lymphocytes (×10^9^/L)	0.44	0.50-4.00
Monocytes (×10^9^/L)	1.30	0.20-1.20
Eosinophils (×10^9^/L)	0.01	0.01-0.50
Basophils (×10^9^/L)	0.04	0.00-0.10
Urea, Serum (mmol/L)	21.2	2.5-7.8
Creatinine, Serum (umol/L)	380	59-104
Potassium, Serum (mmol/L)	4.6	3.5-5.3
Sodium, Serum (umol/L)	122	133-146
C-reactive Protein (mg/L)	144	0-5
Total Protein, Serum (g/L)	74	60-80
Albumin, Serum (g/L)	36	35-50
Alkaline Phosphatase (IU/L)	263	30-130
Alanine Aminotransferase (U/L)	11	0-45
Bilirubin, Total (umol/L)	24	<21
Prothrombin Time (Seconds)	31.5	10.1-11.6
APTT (Seconds)	46.7	20.0-27.0
Fibrinogen (g/L)	4.5	1.9-4.0

With the CT findings in mind, a portable transthoracic echocardiogram (echo) was done one day after his admission. It showed moderate pericardial effusion, normal left ventricular systolic function, impaired right ventricular systolic function, bi-atrial dilatation and patent bioprosthetic aortic valve. Given the blood test results, a further transoesophageal echocardiogram was performed 13 days later. It showed no vegetations and a clean left atrial appendage.

Despite the echo showing no evidence of vegetations, he was diagnosed with infective endocarditis due to multiple splinter haemorrhages, peripheral microemboli and multiple positive blood cultures for *S. haemolyticus*.

Outcome

Despite medical treatment, the patient progressively deteriorated. He passed away from sepsis secondary to *S. haemolyticus* bacteremia 26 days after his admission.

## Discussion

Atherosclerosis is a well-known cause of thrombosis as it is the underlying reason for nearly all causes of coronary artery disease and peripheral arterial disease and many cases of stroke [9]. Additionally, sepsis and infective endocarditis are both well-known causes of thrombosis [6-10]. It can be argued that the renal artery thrombosis in this case occurred due to either the hypercoagulable state induced by sepsis or septic embolism secondary to infective endocarditis in combination with atherosclerosis [11].

The main complaint of the patient was abdominal pain, which has multiple causes that are far more common than renal artery thrombosis [1-4]. While in this case the thrombosis was discovered in the emergency department on the same day of admission, the patient had already developed right renal infarction as he only came to the hospital two days after the onset of his symptoms. Therefore, vascular intervention was not possible.

## Conclusions

This case highlights the diagnostic and therapeutic complexity of renal artery thrombosis occurring during anticoagulation, particularly in the context of sepsis, infective endocarditis and the presence of prosthetic valves. It demonstrates the importance of prompt diagnosis due to the small window during which intervention would be possible.

Sepsis and infective endocarditis can significantly increase thrombotic risk, especially in patients with atherosclerosis. Early multidisciplinary involvement is crucial to optimise outcomes in such rare yet life-threatening presentations.
